# Evaluating the Practicality of Causal Inference From Non-randomized Observational Data in Small-Scale Clinical Settings: A Study on the Effects of Ninjin’yoeito

**DOI:** 10.7759/cureus.55825

**Published:** 2024-03-08

**Authors:** Nobuo Okui

**Affiliations:** 1 Dentistry, Kanagawa Dental University, Kanagawa, JPN

**Keywords:** propensity score matching, difference-in-differences, instrumental variable, inverse probability of treatment weighting, overactive bladder symptom score, ninjin'yoeito, non-randomized observational data, causal inference

## Abstract

Objective

The primary goal of this study was to demonstrate the practical application of causal inference using non-randomized observational data, adapting this approach to smaller populations, such as those in hospitals or community healthcare. This adaptation seeks a more effective and practical research method than randomized controlled trials (RCTs), with the goal of revealing novel insights unexplored by traditional research and enhancing understanding within the realm of causal inference.

Methods

This study evaluated the effects of Ninjin'yoeito (NYT), a traditional Japanese Kampo medicine, on Overactive Bladder Symptom Score (OABSS) and the frailty scores. Employing new statistical methods, this study sought to illustrate the efficacy of estimating causal relationships from non-randomized data in a clinical setting. The database included 985 women aged 65-90 years who visited a clinic between November 2016 and November 2022. By utilizing various statistical techniques, including regression analysis, inverse probability of treatment weighting (IPTW), instrumental variable (IV), and difference-in-differences (DiD) analysis, this study aimed to provide insights beyond traditional methods, attempting to bridge the gap between theory and practice in causal inference.

Results

After applying propensity score matching, the NYT treatment group (220 participants) and non-treatment group (182 participants) were each adjusted to two groups of 159 individuals. NYT significantly improved OABSS and frailty scores. IPTW analysis highlighted that on average, the NYT treatment group showed an improvement of 0.8671 points in OABSS and 0.1339 points in the frailty scores, surpassing the non-treatment group (p<0.05). IV analysis indicated that NYT treatment is predicted to increase ΔOABSS by an average of approximately 4.86 points, highlighting its significant positive impact on OABSS improvement. The DiD analysis showed that the NYT treatment group demonstrated an average improvement of 0.5457 points in OABSS, which was significantly higher than that of the control group. The adjusted R² value for the model is 0.025.

Conclusion

This study successfully implemented a practical application of causal inference using non-randomized observational data in a relatively small population. NYT showed a significant improvement in OABSS and vulnerability, and this result was confirmed using a new statistical method. The relatively low adjusted R² of the model suggests the existence of other unmeasured variables that influence OABSS and vulnerability improvement. In particular, the use of diverse statistical techniques, including IPTW, IV, and DiD analysis, is an important step toward revealing the effectiveness of inferring causal relationships from non-randomized data and narrowing the gap between theory and practice. This study provides a valid and practical alternative to RCTs and reveals new insights that have not been explored in traditional research.

## Introduction

Randomized controlled trials (RCTs) are recognized as the gold standard for establishing causal relationships in medicine and employ experimental methods to assess new treatments with participants randomly assigned to different intervention groups [1,2]. However, ethical concerns, real-world clinical setting differences, sample size, and bias present challenges that necessitate observational study data analysis [1,2].

Owing to ethical and logistical constraints, RCTs of drugs and surgical treatments tend to face many obstacles, making observational studies using causal inference an important alternative. Causal inferences in real clinical settings can be adapted through a combination of statistical methods, catering to the analysis of comparatively smaller populations [3-8]. This is particularly relevant for older adults with frailty who are more likely to drop out of RCTs, highlighting the need for a multifaceted analysis of observational data [9]. Traditional Japanese Kampo medicines, affected by diverse factors and known for their varied effects, underscore the significance of using observational data to establish causality between health and medicine [9].

This paper outlines the fundamental methods for estimating advanced causal relationships using non-randomized observational data for smaller populations [5,6]. Specifically, this study focuses on causal inference methods using multiple statistical models, including logistic regression for propensity scores [7,8], inverse probability of treatment weighting (IPTW), augmented difference-in-differences (DiD) analysis, and instrumental variable (IV) analysis [5-8]. While earlier statistical methods suggested NYT's efficacy for frailty and the potential for an overactive bladder (OAB), advanced causal inference provides a more accurate analysis of NYT's effects of NYT on OAB, identifying further areas for investigation [9]. This study demonstrates that causal inference is applicable and beneficial, even for relatively small populations, encouraging researchers to understand basic causal inference strategies and further explore this field.

## Materials and methods

Ethics committee and patient data

This study was approved (No. 24-002) by the Ethics Committee of the Yokosuka Urogynecology and Urology Clinic and utilized a database from a prior study to examine the effects of drug treatments on a small population through causal inference. The population and questionnaires used in a previous study were also employed. The objective is to verify whether the same results can be obtained with this database using several statistical methods for causal inference and to discover new insights. Typically, statistical studies use data that have already produced results or dummy data. In this study, we used the data from a previous study. The details are as follows.

The subjects of the data collected in the previous study were women aged between 65 and 90 years who visited our clinic between November 2016 and November 2022 [9]. All participants provided informed consent before their inclusion in the study. Using these data allows for verification with higher precision than previous studies and the possibility of identifying any deficiencies.

Data collection

At their initial consultation, patients completed multiple assessments under the supervision of a nurse, including the frailty scores (FRAILTY), the International Consultation on Incontinence Questionnaire-Short Form, and the Overactive Bladder Symptom Score (OABSS) [9]. A physician evaluated the Vaginal Health Index Score and conducted a vulvar pain swab test [9]. Frailty is typically defined as a clinical syndrome characterized by a decrease in strength, endurance, and physiological function that increases an individual's vulnerability to developing increased dependency and/or death [9]. The concept of frailty often includes components, such as fatigue, resistance, ambulation, illnesses, and weight loss. OAB is defined as a condition characterized by symptoms such as urinary urgency with or without urge incontinence, usually with frequency and nocturia [9].

Baseline and follow-up evaluation

The baseline (T0) was set as the first visit for patients who had previously been evaluated for OAB or were currently taking OAB medication. If newly evaluated, T0 was designated one month after the assessment and initiation of the appropriate OAB medication. The primary evaluation point was 12 months (T12) when the state of frailty was determined using the frailty scores at T0. Based on treatment preferences, patients were divided into two groups: those who received NYT for 12 months (treatment regimen) and those who did not receive NYT. The patients were administered 7.5 g/day of NYT granules (KB-108, Kracie Pharma Ltd., Tokyo, Japan). This medication was covered by insurance, and the dosage followed the recommendations of the Pharmaceuticals and Medical Devices Agency [9].

Eligibility criteria

The eligibility criteria specified women aged 65 years and older, corresponding to the American Society of Anesthesiologists Physical Status Classification of I-III. Patients with a follow-up period of less than one year or those with stage 2 or higher pelvic organ prolapse were excluded.

Causal inference research methods

In this study, causal inference was conducted based on the Rubin Causal Model [10]. The objective of this study was to evaluate the effect of NYT treatment on the improvement of OABSS and FRAILTY. Specifically, the analysis focuses on the improvement rates (ΔOABSS and ΔFRAILTY). ΔOABSS is defined as the difference in OABSS scores between pre-treatment (T0) and 12 months post-treatment (T12), whereas ΔFRAILTY represents the difference in FRAILTY between the same time points. Multiple statistical analysis methods were used for evaluation.

Regression analysis

In the regression analysis, ΔOABSS and ΔFRAILTY were used as dependent variables, and the presence of NYT treatment (independent variable), along with patient characteristics such as age, BMI, and grip strength (covariates), were considered in a multivariate regression analysis. This analysis assessed whether the presence of treatment significantly influenced the improvement rates. ΔOABSS and ΔFRAILTY were compared between the NYT treatment group (NYT group) and control group (non-NYT group).

**Figure 1:**
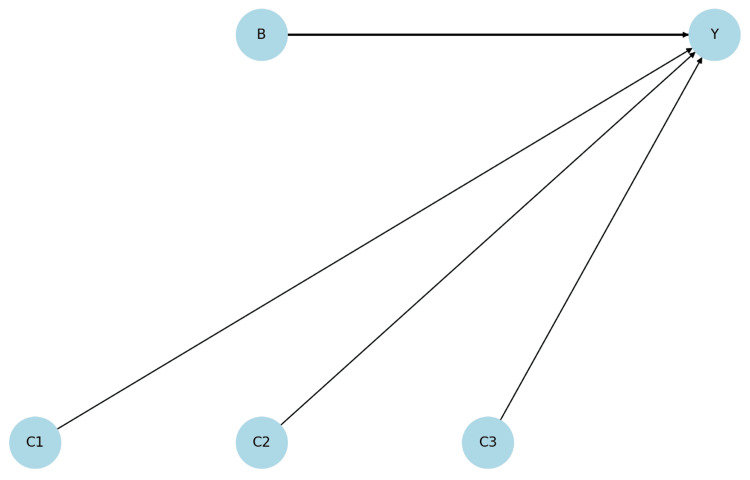
Directed Acyclic Graph Illustrating the Causal Relationship for ΔOABSS B: Instrumental Variable (Behavioral Variable/Intervention Variable) Y: Outcome Variable (Y Variable) C1: Covariate 1 (Age) C2: Covariate 2 (BMI) C3: Covariate 3 (Grip Strength) ΔOABSS: The improvement rate in OABSS, calculated as the difference between post-treatment and pre-treatment OABSS, OABSS: Overactive Bladder Symptom Score, BMI: Body Mass Index

Figure 1 shows a Directed Acyclic Graph (DAG) illustrating the causal relationship of ΔOABSS [11]. This DAG visually represents how an instrumental variable (B) affects ΔOABSS (Y). Although the effects of covariates (C1, C2, C3) on the outcome variable are not statistically significant, they do exert some influence. This also indicates that the instrumental variable does not affect the covariates (exogenous), which enhances the reliability of this analysis. The diagram hypothetically varies the thickness of the edges, assuming that B and Y are significant, while C1, C2, and C3 are not significant with Y.

IPTW Analysis

We estimated the probability of treatment assignment based on patient characteristics (i.e., propensity score). By weighing each patient based on this score, we adjusted for baseline characteristics between the treatment and control groups. This approach mitigates selection bias in observational data and enhances the accuracy of the treatment effect estimation. IPTW analysis is a method for a more accurate estimation of the effect of treatment, taking into account biases in treatment assignment [12,13]. In this study, the IPTW method was used to evaluate the effect of NYT treatment on ΔOABSS and ΔFRAILTY.

**Figure 2:**
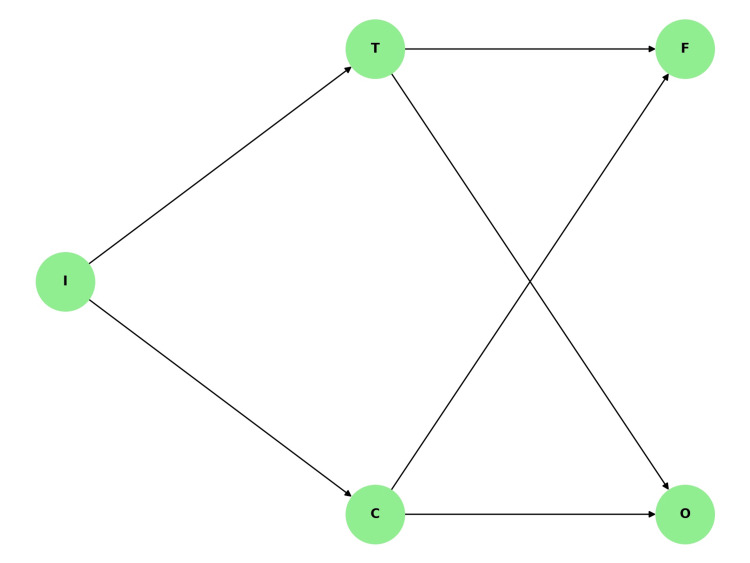
Directed Acyclic Graph Illustrating Causal Relationships Based on IPTW Analysis Covariate I: IPTW (Inverse Probability of Treatment Weighting) T: Treatment Group (NYT Treatment) C: Control Group O: ΔOABSS F: ΔFRAILTY ΔOABSS: The improvement rate in OABSS, calculated as the difference between post-treatment and pre-treatment OABSS, OABSS: Overactive Bladder Symptom Score, ΔFRAILTY: The improvement rate in FRAILTY, calculated as the difference between post-treatment and pre-treatment FRAILTY, NYT: Ninjin'yoeito

Figure 2 visually depicts the transition from the IPTW method to subsequent IV analysis. The IPTW method was adjusted for baseline characteristics between the treatment group (T) and the control group (C), reducing selection bias in observational data. It also illustrates the impact pathways from the treatment group to ΔOABSS and ΔFRAILTY (O and F), thereby enhancing the accuracy of the treatment effect estimation. This figure complements the concept of IPTW analysis and is positioned to deepen the understanding of the IV analysis.

IV analysis

We used IV analysis to address the biases stemming from the endogeneity of treatment assignments [14]. This method is particularly useful in situations in which the assignment of treatment is not random. In this study, IV analysis was used to estimate the effects of the NYT treatment.

Specifically, the presence or absence of antimuscarinics, β3-adrenoceptor agonists, and a combination of drugs were utilized as instrumental variables to more accurately estimate the effects of NYT treatment.

DiD analysis

The DiD analysis estimated the effect of a specific intervention (NYT treatment) by comparing the changes in improvement rates before and after the intervention between the treatment and control groups [15-17]. This analysis isolates and evaluates the pure effect of the intervention, taking into account external influences, based on the assumption that in the absence of the intervention, the treatment and control groups would follow the same trend. Specifically, outcome data were collected before and after the intervention, and the average change for each group was calculated. The difference in the average change between the treatment and control groups was determined, and the resulting DiD represented the effect of the intervention.

Model fit

Model fit is a crucial metric for quantitatively assessing how well a statistical model fits the observed data [18,19]. The adjusted R² is an indicator that shows the proportion of variance in the data explained by the model. R² values range from 0 to 1, with values closer to 1 indicating a higher degree of model fit. The F-statistic was used to evaluate the overall statistical significance of the models. It tests whether the explanatory variables in the model explain the variance in the dependent variable statistically significantly. A larger F-statistic implies that the explanatory variables better explain the variation in the dependent variable, and is usually reported alongside the corresponding p-value.

Analysis procedure

The test was conducted in four stages to minimize potential biases in the evaluation of treatment effects and to produce more reliable results: (1) Data collection: Data on the improvement rates in OABSS and FRAILTY, as well as age, BMI, and grip strength, were collected from both the patient group that received NYT treatment and the untreated control group; (2) Estimation of propensity scores: Using logistic regression, we estimated the propensity scores for each patient to receive NYT treatment; (3) Construction of statistical models: Multiple statistical models, diagnosis of multicollinearity, IPTW, IV analysis, and augmented DiD analysis, were constructed; (4) Estimation and testing of effects: Using each statistical model, we estimated the effects of NYT treatment on OABSS and FRAILTY, and tested for statistical significance.

Statistics

Statistical analyses were conducted and confirmed using Python code (Python Software Foundation, Wilmington, Delaware, United States) supported by ChatGPT4.0 (OpenAI, San Francisco, California, United States). These processes were executed on a Windows 10, version 1903 operating system (Microsoft Corporation, Redmond, Washington, United States). Statistical significance was determined using a p-value < 0.05.

## Results

Patients

In previous studies, among 985 outpatients seen at our clinic throughout the study period, 725 were identified as frailty/pre-frailty and 260 as non-frailty based on their FRAILTY. Of these, 402 frail/pre-frail women (average age 77.5 ± 6.49 years) satisfied the study's eligibility requirements, with a median follow-up duration of 14.5 months. Initially, there were 220 women in the NYT group and 182 in the non-NYT group, and propensity score (PS) matching was equalized to 159 women per group. In the database and not previously displayed in the publication from these studies, we have the following information: The average BMI was 23.71 ± 3.43 kg/m² in the NYT group and 22.82 ± 4.25 kg/m² in the non-NYT group (p=0.051). Regarding grip strength, the averages were 17.49 ± 3.87 kg for the NYT group and 16.87 ± 4.24 kg for the non-NYT group (p=0.128).

Multiple statistical model results

Compared to the non-NYT group, the NYT group had a significantly higher average ΔOABSS by 0.4748 points (p = 0.002). Age (p=0.519) and grip strength (p=0.611) did not have a significant impact on OABSS improvement. The adjusted R² was 0.030 and the F-statistic was 4.041 (p=0.00318), indicating that the model was statistically significant.

Moreover, the NYT group showed a significantly higher average ΔFRAILTY (0.1275 points) than the non-NYT group (p < 0.0001). Age was significantly associated with improvement in FRAILTY (p=0.003). BMI and grip strength did not significantly affect the improvement in the FRAILTY score (p > 0.05). The adjusted R² was 0.055 and the F-statistic was 6.841 (p=2.48e-05), indicating that the model was statistically significant.

**Figure 3:**
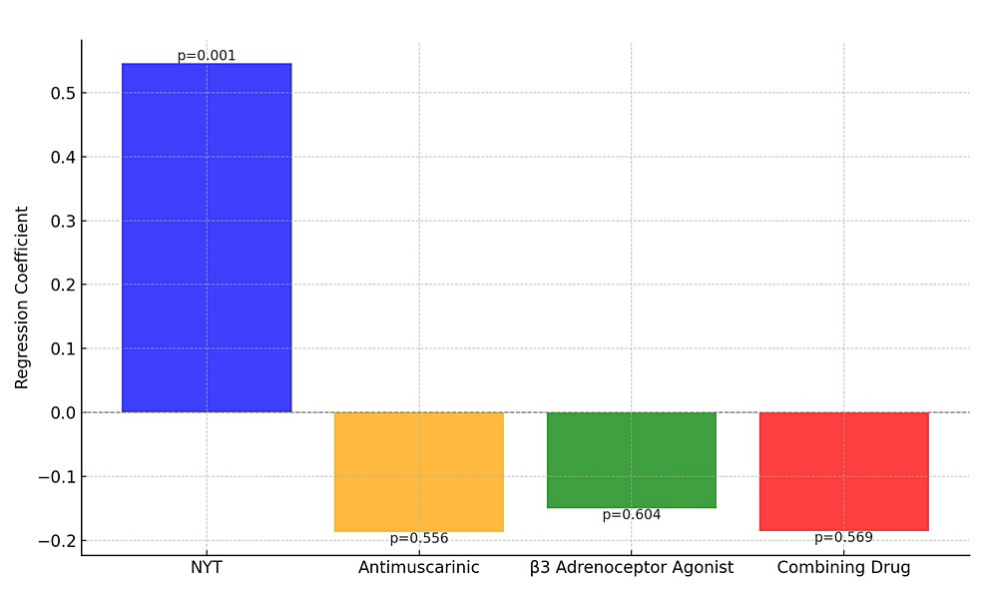
Regression Coefficients and Their Statistical Significance Vertical Axis (Y-axis): Regression Coefficients, Unit: Points Horizontal Axis (X-axis): Treatment and Medication Groups (NYT Treatment, Blue; Antimuscarinic: Yellow; β3 Adrenoceptor Agonist, Green; Combining Drug, Red), NYT: Ninjin'yoeito Numbers above bars indicate the p-values, representing the statistical significance of the regression coefficients for each treatment group

Figure 3 displays the regression coefficients and their statistical significance for the change in the OABSS across various treatments and interventions. The blue bar represents the coefficient for the NYT group, indicating a statistically significant positive effect with a p-value of 0.001. In contrast, the orange bar for antimuscarinics, the green bar for β3 adrenoceptor agonists, and the red bar for combining drug treatments show p-values of 0.556, 0.604, and 0.569, respectively, indicating that these treatments are not statistically significant. These results suggest that NYT treatment may have a statistically significant effect on improving the symptoms of overactive bladder, whereas the other treatment groups and drug combinations do not show such clear effects.

Diagnosis of multicollinearity results

Prior to conducting the regression analysis, a diagnosis of multicollinearity was performed to ensure that excessive correlations among variables did not introduce bias into the results. The Variance Inflation Factor (VIF) measures the degree of correlation between independent variables, and values exceeding 10 are indicative of high multicollinearity.

**Figure 4:**
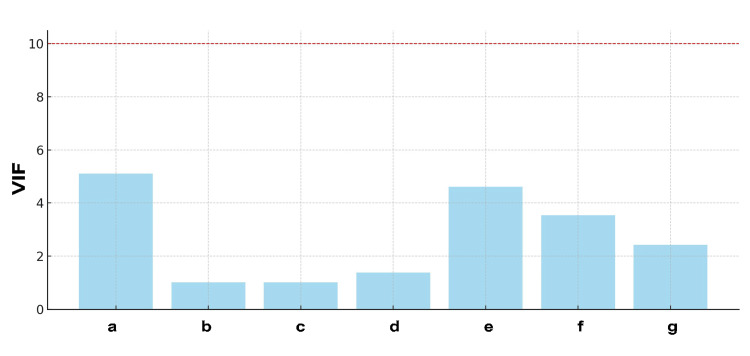
Assessment of Multicollinearity Using VIF Y-Axis Label: VIF (No units) X-Axis Label: Predictors (Coded) a: Age, b: BMI, c: Grip Strength, d: NYT, e: Antimuscarinic, f: β3 Adrenoceptor Agonist, g: Combining Drug NYT: Ninjin'yoeito, VIF: Variance Inflation Factor The red dashed line at the top represents a VIF threshold, above which multicollinearity might distort the regression analysis

Figure 4 presents the results of the VIF analysis. Contrary to what was previously mentioned, the highest VIF value was observed for age (VIF ≈ 5). The VIF values for antimuscarinic and β3 adrenoceptor agonists were lower than initially stated, at approximately 4 and 3.5, respectively. However, because all these values were below the commonly accepted threshold of 10, the level of multicollinearity in the model was considered to be within acceptable limits. Therefore, these variables were retained in the model. The VIF values for BMI (VIF ≈ 2), grip strength (VIF ≈ 1.5), NYT (VIF ≈ 3), and combination drugs (VIF ≈ 4) were lower, suggesting no concerning bias based on their correlations.

IPTW analysis results

In the analysis using the IPTW method, the improvement rate in OABSS for the NYT treatment group was, on average, 0.8671 points higher than that of the control group, with this difference being statistically significant at a p-value of 0.0004. For the improvement rate in FRAILTY, the NYT treatment group showed an average increase of 0.1339 points compared to the control group, with a statistically significant difference at a p-value of 1.08e-06. IPTW analysis enabled the estimation of improvement rates adjusted for selection bias, thereby enhancing the reliability of the treatment effect assessment.

**Figure 5:**
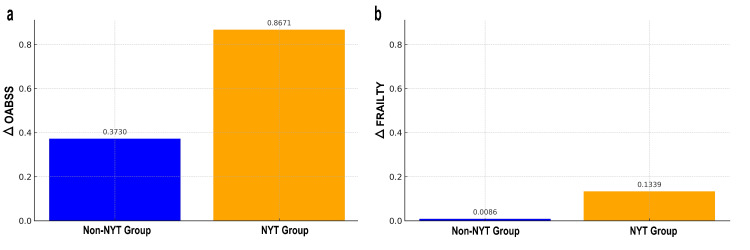
Weighted Average Improvement Rates for OABSS and FRAILTY a: ΔOABSS, b: ΔFRAILTY Vertical axis: Weighted average improvement rate, unitless Horizontal axis: 0 represents the non-NYT group (non-treatment, blue bar), 1 represents the NYT treatment group (with treatment, orange bar) The numerical values displayed above each bar represent the specific improvement rates for each group ΔOABSS: The improvement rate in OABSS, calculated as the difference between post-treatment and pre-treatment OABSS, OABSS: Overactive Bladder Symptom Score, ΔFRAILTY: The improvement rate in FRAILTY, calculated as the difference between post-treatment and pre-treatment FRAILTY, NYT: Ninjin'yoeito

Figure 5 shows the weighted mean improvement rates for ΔOABSS and ΔFRAILTY after adjustment using the IPTW analysis. For OABSS, the weighted mean improvement rate for the non-NYT group (blue bars) is 0.3730, while for the NYT group (orange bars) it is 0.8671, indicating that the group receiving NYT treatment exhibited a higher improvement. Regarding FRAILTY, the weighted mean improvement rate for the non-NYT group was 0.0086, and for the NYT treatment group, it was 0.1339, which also showed a higher improvement rate for the NYT treatment group. These results demonstrate a significant difference between the NYT treatment and Non-NYT groups in terms of improvement rates for both OABSS and FRAILTY, confirming the effectiveness of NYT treatment.

IV analysis results

In the ΔOABSS, the coefficient for the predicted allocation of NYT treatment (an estimate of treatment effect) is approximately 4.8594, indicating that when NYT treatment is predicted, ΔOABSS increases by an average of about 4.86 points (with an intercept of approximately -2.0543). The results of the IV analysis suggest that NYT treatment has a significant positive impact on the improvement of the OABSS. This analysis allows for an estimation of the treatment effect, considering the endogeneity bias of treatment allocation, providing more reliable evidence for the effectiveness of NYT treatment.

DiD analysis results

Compared to the control group, the NYT treatment group showed an average improvement in OABSS of 0.5457 points, which was statistically significant (p = 0.001). The use of antimuscarinic, β3 adrenoceptor agonist, and a combination of drugs did not have a statistically significant impact on the improvement rate of OABSS (p > 0.05). The analysis results indicate that considering endogeneity bias and other potential confounding factors, NYT treatment has a significant effect on the improvement of OABSS and FRAILTY, providing important information for the selection of treatment.

Impact of additional variables

The analysis results indicated that age did not have a statistically significant effect on the improvement rate of the OABSS (p > 0.05). Similarly, BMI did not have a statistically significant effect on ΔOABSS (p=0.110).

Model fit

The adjusted R² for the model used in this study was 0.025, which is relatively low, indicating that the model explained only 2.5% of the overall variance in the data. This suggests that there may be other unmeasured variables that could potentially affect the improvement of the OABSS and FRAILTY, highlighting the need for further research. The F-statistic is 2.466 with a p-value of 0.0174, which confirms that the overall model is statistically significant, suggesting that the variables included in the analysis collectively have a meaningful impact on ΔOABSS. The analysis results support the significant effect of NYT treatment on the improvement of OABSS and FRAILTY. At the same time, the lack of a statistically significant impact from additional variables such as age, BMI, and grip strength on ΔOABSS indicates that the effects of the treatment itself may be more consequential than these factors.

## Discussion

Both this study and previous research have demonstrated the effectiveness of Ninjin'yoeito (NYT) against OAB. Previous research utilized a t-test to compare the two groups after matching observational data with propensity scores, which showed significant improvements in ΔOABSS (p=0.001) with NYT. By contrast, this study employed more advanced statistical methods to minimize potential biases and enhance the reliability of causal effect estimation, proving NYT's impact on OABSS and identifying an issue with a model fit (R² = 0.025). The techniques used in this research are part of what is known as advanced causal inference research, which is highly valued in fields such as policy evaluation, economics, medical research, and social science. This approach provides more reliable scientific evidence in situations where randomized controlled trials (RCTs) are impossible or inappropriate [20,21].

Although research on causal inference using observational data has attracted attention, its use in clinical medicine is limited. This is believed to be because advanced causal inference research requires a combination of complex statistical models, which can be a barrier to many clinical medical researchers. Next, we examine the statistical methods used in this study, along with the evidence [20,21].

In this study, multiple approaches, including propensity score, IPTW, IV analysis, and extended DiD analysis, were employed for statistical analysis. These methods were instrumental in analyzing the impact of NYT treatment on the improvement of OABSS and FRAILTY scores among women aged 65 years and older using observational data. Each method corroborated the significant effect of NYT in improving OAB and frailty.

Propensity score matching is a crucial method in causal inference based on observational data for adjusting confounders between the treatment and control groups, thereby creating comparable groups. Liang and Liu reported that this approach plays a significant role within the framework of the Rubin causal model, reducing the bias in treatment effects [22]. Propensity score matching provides consistent outcomes without the assumption of interference, aids in mitigating the impact of unmeasured confounders, and enhances the reliability of results. However, Kane et al. pointed out the limitations of propensity score matching in surgical research [3,23].

In this study, we used propensity score matching to ensure that one person's treatment did not affect another, meeting the no-interference assumption. The results between the treated and untreated groups were consistent, indicating that our method accurately reflected the real outcomes. We also used an IV analysis to reduce the effect of any unmeasured factors, although we could not eliminate them completely. By evenly splitting the participants into treatment and control groups, we partially met the positivity assumption, indicating a fair chance of treatment for everyone and supporting the reliability of our findings.

The IPTW method was developed to compensate for the shortcomings of propensity scores, with the aim of balancing the treatment and control groups to facilitate the inference of causal relationships. Allan et al. reported that weights, which are the inverse of propensity scores, adjust for differences, and that modified weights improve stability [24]. Austin and Stuart evaluated baseline balance using IPTW, compared it with other methods, and found that IPTW was effective in causal inference [12].

In this study, IPTW method analysis indicated that age did not significantly impact the OABSS improvement rate (p > 0.05). Although BMI did not show a statistically significant effect on the improvement rate, its p-value was very close to the commonly accepted threshold for statistical significance (p < 0.05) at 0.051. This suggests that BMI may have an influence on the improvement rate of OABSS, but the effect was not statistically significant. These results imply that the efficacy of the treatment itself is more important than factors such as age or BMI in the selection of interventions. However, the possibility that BMI may affect treatment outcomes cannot be completely ruled out.

IV analysis was developed to overcome endogeneity bias from unmeasured confounders affecting both treatment and outcome, whereas IPTW analysis was adjusted for selection bias. However, as Hogan and Lancaster pointed out, these methods do not entirely eliminate bias, necessitating caution when interpreting the results [25]. In particular, IV analysis is subject to potential bias in the estimates if the assumption that the instrumental variable is completely exogenous is not met. Furthermore, Gruber et al. highlighted several limitations in the application of IPTW and IV analysis, including reliance on positive assumptions, the need for finite IP weights in practical applications, and the requirement for Targeted Maximum Likelihood Estimation (TMLE) for propensity scores to be away from 0 and 1 [26]. These considerations underscore the complexity and challenges of applying these statistical methods to derive unbiased causal inferences from observational data.

In this study, the application of the IPTW method and IV analysis statistically confirmed that NYT treatment significantly improved OABSS by an average of 0.8671 points (p=0.0004) and FRAILTY by 0.1339 points (p = 1.08x10^-6^). The IV analysis estimated the predicted increase in ΔOABSS due to NYT treatment to be approximately 4.86 points. These findings indicate that the chosen statistical methods enhance the reliability of the treatment-effect estimates and improve the accuracy of causal inference. This approach underscores the importance of using advanced statistical techniques to validate the efficacy of interventions in observational studies, particularly when RCTs are infeasible.

A DiD analysis evaluates the effect of interventions over time by comparing changes in outcomes between the treatment and control groups before and after the intervention. Stuart et al. proposed a new method that uses DiD models to estimate the effects of policy changes and improve the balance of characteristics between the intervention and comparison groups over time [27]. Conducting IPTW and IV analyses beforehand enhances the comparability between treatment and control groups, reducing the influence of endogeneity bias and unmeasured confounders. This prepares the grounds for employing DiD analysis to assess the effect of interventions over time more accurately [28].

In this study, the results of the DiD analysis indicated that the NYT treatment group showed a statistically significant improvement in OABSS, with an average increase of 0.5457 points compared to the control group. It was also shown that the use of antimuscarinics, β3 adrenoceptor agonists, and a combination of these drugs did not have a statistically significant impact on the improvement rate of OABSS. Considering endogeneity bias and other potential confounding factors, the analysis results demonstrate that NYT treatment has a significant effect on the improvement of OABSS and FRAILTY, providing important information for treatment selection.

Finally, the summary highlights recent discoveries on issues with RCTs and causal inferences from non-randomized observational data. Limitations of RCTs, as Mulder et al. pointed out, include their inability to forecast efficacy in actual clinical scenarios [29]. Harrer et al. highlighted both evitable and intrinsic challenges in RCT design within mental health research, noting biases and power deficiencies [30]. Crane et al., among others, showed how observational studies provide dependable causal insights, notably in areas such as obesity prevention where RCTs prove unviable [31]. The critical role of meticulous design in observational studies on rare diseases was emphasized by Izem and McCarter, while Sourial et al. advanced causal inference research by tackling biases and refining methodological strategies [32,33]. Furthermore, evidence suggests that statistical methods can address biases in observational data, indicating their extensive applicability in clinical research but also warning about the inherent limitations of observational studies. These insights encourage the incorporation of observational data into forthcoming research and the advancement of causal inference methodologies, making a substantial contribution to the discipline.

This study had three main limitations. First, even with the use of advanced statistical techniques to analyze observational data, there is an inherent risk of unmeasured confounders that could affect the outcomes. Second, the model's explanatory power (adjusted R² = 0.025) was valid but relatively low. A potential solution could involve exploring Hernán's targeted trial framework [34]. Finally, the causal inference techniques used in this study are not universally applicable to all observational studies. Analyzing observational data for causal inferences requires a combination of multiple statistical methods.

## Conclusions

This study evaluated the practicality of causal inference using non-randomized observational data and explored its application in real-world clinical practice, especially in relatively small populations. By combining advanced statistical techniques such as inverse probability of treatment weighting, instrumental variable, and difference-in-differences analysis, we were able to demonstrate significant effects on overactive bladder and frailty with greater precision than in previous studies. Additionally, the relatively low adjusted R² of the model indicated the presence of other unmeasured variables that influenced Overactive Bladder Symptom Score and vulnerability improvement.

These results offer a new perspective on the application of causal inference from non-randomized observational data in real-world clinical settings as a practical research method and alternative to randomized controlled trials. These findings highlight the importance and potential of causal inference research for practical and direct clinical applications, encouraging the adoption of innovative methodological approaches in future research.
